# Mediating effects of social support and presenteeism on turnover intention and post-traumatic stress disorder among Chinese nurses in the post-pandemic era: a cross-sectional study

**DOI:** 10.3389/fpubh.2024.1323126

**Published:** 2024-02-14

**Authors:** Jingshuo Zhang, Xin Yang, Xiaoman Zhang, Yuping Liu, Mengshi Liu, Yu Fang, Mengjie Liu, Min Wu

**Affiliations:** ^1^School of Nursing, Xuzhou Medical University, Xuzhou, Jiangsu, China; ^2^The Affiliated Hospital of Xuzhou Medical University, Xuzhou, Jiangsu, China

**Keywords:** turnover intention, post-traumatic stress disorder, social support, presenteeism, COVID-19

## Abstract

**Background:**

The shift in national policies for epidemic prevention and control in the post-pandemic era is undoubtedly a challenge for health care professionals. Nurses, as an important part of the health care professional population, have an even greater impact on their mental health and occupational safety. This may expose nurses to post-traumatic stress disorder (PTSD) and presenteeism, and ultimately lead to their turnover.

**Objective:**

This study aimed to investigate the relationship between turnover intention and post-traumatic stress disorder among Chinese nurses during post-pandemic era, and the mediating role of social support and presenteeism.

**Methods:**

In this study, a multicentre cross-sectional survey was conducted in April 2023 among nursing staff in several tertiary general hospitals in northern China, with online data collection using the Turnover intention Scale (PTSD), the Impact of Events Scale (IES), the Social Support Scale (SSS), and the Stanford presenteeism Scale (STAS) and the relationship between variables was analyzed using hierarchical multivariate regression, and Structural Equation Modeling (SEM) was used to analyze the relationship between post-traumatic stress disorder and the Turnover intention from the pathway between.

**Results:**

Data were collected from 2,513 nurses who met the inclusion criteria, in which general information such as age, department, specific department, Professional title, history of alcohol consumption, form of employment, Years of working, and Average working hours per day were statistically significant with the difference in the turnover intention. The results of the study showed a 32% high turnover intention among nurses in the post-pandemic era, which was lower than the turnover intention during the pandemic. The results of hierarchical multiple regression analysis showed that post-traumatic stress disorder, social support, and presenteeism were significant predictors of turnover intention. The total effect of post-traumatic stress disorder on turnover intention to work was 0.472 [bias modified 95% confidence interval (0.415–0.483), *p* < 0.001]. Social support and attendance played a partially intermediate role in post-traumatic stress disorder and propensity to leave (an indirect effect of 26% of the total effect).

**Conclusion:**

Turnover intention and post-traumatic stress disorder levels are high and social support plays an important role in the tendency to leave the job and post-traumatic stress disorder, healthcare institution can be achieved by strengthening social support for nurses in the post-epidemic era and preventing the occurrence of presenteeism.

## 1 Introduction

In recent years, frequent global outbreaks of pandemic have become a serious challenge for the world (1, 2). COVID-19 pandemic, as a public health emergency, has had a profound impact on the global health system and socio-economics (3, 4). As an important part of the front-line of the fight against epidemics, looking at the infection rate among healthcare professionals, nurses had the highest degree of infection, which can be explained by direct contact with patients (1, 5). In the context of sudden onset epidemics, some studies have shown that nurses are not only under immense pressure to perform their medical tasks, but they also have to cope with a variety of challenges, such as psychological stress, risk of infection, and personal life-work balance (6).

The transition to the post-epidemic era has brought about a notable rise in the number of infected patients (7). However, challenges such as a shortage of medication and insufficient medical resources have exacerbated the situation. Consequently, healthcare workers are facing extended working hours and considerable psychological pressure, leading to severe mental health problems among them.

Post-traumatic stress disorder (PTSD) is a mental health condition that can develop after a person experiences or witnesses a traumatic event (8). It is characterized by a range of symptoms that persist long after the traumatic event. During the epidemic, there was research that revealed a complex trajectory of PTSD symptoms over time. Nurses' frequent handling of traumatic events and high-pressure work environments may lead to burnout, which increases the risk of burnout and resignation (9, 10). This may lead to turnover and a lack of senior nurses in the nursing profession (11). One piece of research on PTSD in nurses during a pandemic suggests that nurses are at a higher risk of developing PTSD and should be given the necessary attention (12). Therefore, after the full liberalization of the epidemic prevention and control policy, there is a greater need to further validate this effect to inform subsequent interventions.

Turnover intention is a serious issue that can have a negative impact on both healthcare organizations and the care industry (13). In the post-epidemic era, nurses are in direct contact with patients and may be more susceptible to exposure to infectious diseases. Due to inadequate or poor protective measures, nurses may fear for their health and safety, which may exacerbate their intention to leave their jobs (14). Previous studies have also shown that higher Turnover intention is associated with higher work stress and frequent night shifts during epidemics (15, 16). A higher Turnover intention in the workforce also reduces the quality of healthcare and nursing care, with serious implications for patient safety (17).

The turnover intention of nurses may be related to their psychological trauma during the pandemic, which further leads to a series of symptoms such as post-traumatic stress disorder (15, 18). In recent years, studies have shown that PTSD in nurses is significantly related to turnover intention on the job (14). Liu et al. (19) found that the higher the PTSD among nurses during an epidemic, the higher their turnover intention in their jobs. However, there is a paucity of research demonstrating the mechanisms linking turnover intention and PTSD during the post-epidemic era, with preliminary evidence between studies that a predictor of experiencing high levels of PTSD is potentially turnover intention (20), but relatively little research has been conducted on examining the mediating effects of the Turnover intention a job and PTSD. Therefore, there is a need to continue to explore potential mediating variables between turnover intention and PTSD under post-pandemic era.

Social support plays an important role in the development and coping process of PTSD (21). It can have a positive impact on an individual's mental health, help mitigate the negative effects of trauma, and promote recovery and adaptation (22). Jobson et al. showed that social support was negatively associated with PTSD symptoms (23). Some scholars also believe that social support plays a protective role in turnover intention (24–26). Some researchers have also proposed that healthcare organizations should encourage healthcare workers to utilize the support and communication of family, friends, and coworkers as their primary coping mechanism to manage the adverse mental health consequences of the COVID-19 pandemic (27, 28). However, presenteeism refers to the need for employees to keep working despite their poor physical or mental health (29, 30). During an outbreak, nurses may choose to persevere with their work out of a sense of professional responsibility and mission, even when faced with high-risk and high-stress environments (31). Presenteeism may make nurses more susceptible to exposure to these traumatic situations, as they may forgo rest and devote themselves to their work. Some studies have shown that presenteeism among nurses was a common problem during pandemics, with higher rates of occurrence (32). Whereas, presenteeism leads to a decrease in the quality of care, an increased risk of anxiety, depression, mood swings, and other mental health problems with further contributing to job burnout and turnover (33, 34). It has also been shown that nurses' presenteeism was positively associated with PTSD (35). Labrague et al. also proposed that healthcare organizations should provide effective leadership and organizational support, which is essential to support the needs of healthcare workers and improve their mental health (36). Thus, social support and attendance play a role in connecting PTSD symptoms and the propensity to leave (37). Nurses with social support may be able to cope more effectively with the psychological stress of the epidemic and reduce PTSD symptoms, thereby reducing the propensity to leave (38, 39). In contrast, those who exhibit attendance may be more able to cope with the psychological stress of the epidemic by being forced to work overtime, exacerbating PTSD symptoms and thus increasing the propensity to leave (40). Notably, previous studies have not further measured the mediating role of social support and presenteeism in the relationship between turnover intention and PTSD symptoms (38, 40, 41).

**Figure 1:**
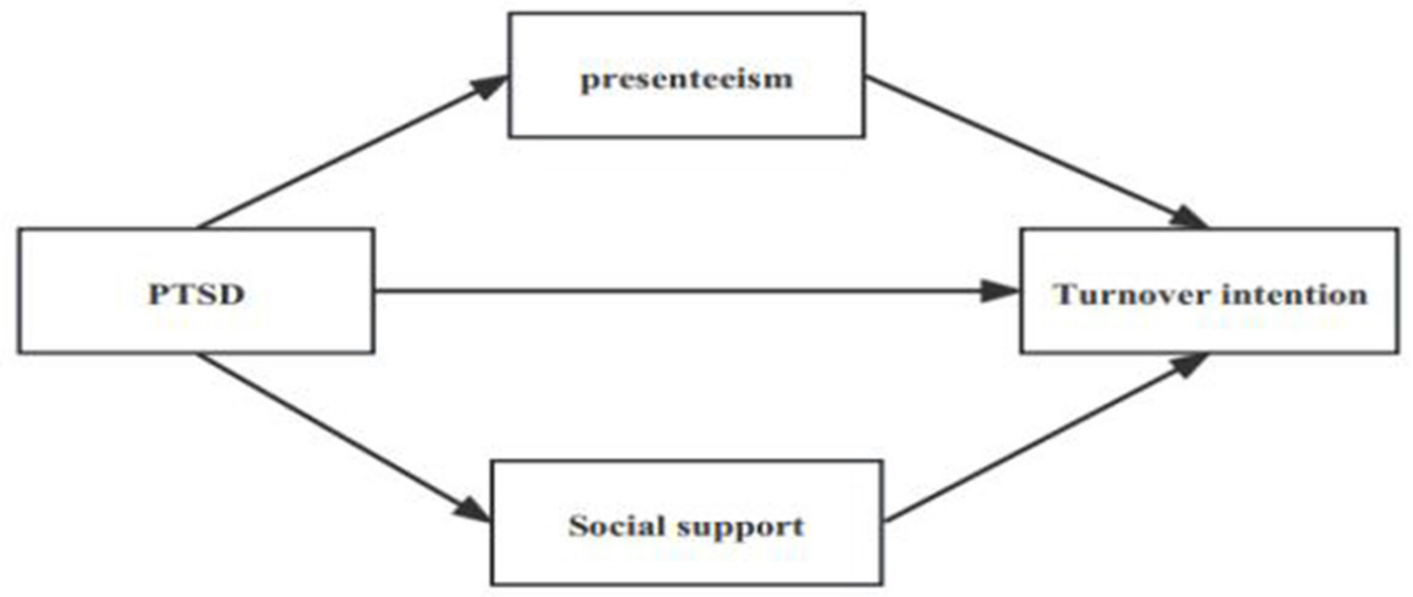
Theoretical model.

Given the combination of previous findings and the existing literature, this study aimed to systematically examine the relationship among PTSD, social support, presenteeism, and turnover intention a job of nurses during the post-pandemic era. The following hypotheses were proposed: (1) PTSD predicts the turnover intention, (2) social support makes a mediates role in the relationship between PTSD and turnover intention, and (3) presenteeism mediates in the relationship between PTSD and turnover intention the job (Figure 1).

## 2 Methodology

### 2.1 Design and participants

In this study, stratified sampling method was used to select three tertiary general hospitals. According to the level of medical development in northern China, we divided them into three layers, and selected nurses from one tertiary general hospital from each layer for investigation. Inclusion criteria: age ≥ 18 years; working in the clinical work during the epidemic; registered nurses with license; willingness to participate in this study. Exclusion criteria: Nurses who were not engaged in work for long periods of leave; nurses with previous mental illness or other serious physical illness. This study followed the STROBE reporting guidelines (42). In this cross-sectional study, the sample size was calculated according to the formula *n* = 4$\mu_{\text{α}}^{2}\sigma^{2}$/δ^2^ for quantitative data (43). According to the results of previous literature (44), the standard deviation of turnover intention is σ = 3.44; the allowable error δ = 1, α = 0.05; considering the shedding rate of 10%, the sample size was determined to be at least 200. The final actual obtained sample content was 2,513.

### 2.2 Measuring tools

#### 2.2.1 General information

It included gender, age, department where they were working, education background, professional title, whether they were an only child, marital status, whether they had a history of alcohol consumption, whether they had taken sedative and sleeping medications, form of employment, years of employment, hours of work per day, specific department where they were working, whether they participated in the provincial or municipal outbreak assistance program, whether they have cared for a patient with the novel coronavirus, the outcome of caring for that patient with the novel coronavirus, whether they have been infected with the novel coronavirus, the number of times they have been infected with the novel coronavirus, and their monthly income.

#### 2.2.2 Turnover intention

The Turnover intention is based on the Turnover Intention Scale, which has a total of three dimensions and six entries (45). Dimension 1: Likelihood of quitting current job, dimension 2: Motivation to find another job, and Dimension 3: Likelihood of obtaining an outside job. Each entry was scored on a 4-point Likert scale, “Never” (1 point), “Rarely” (2 points), “Occasionally” (3 points), and “Frequently” (4 points), with a total scale score of 6–24, with higher scores representing a higher likelihood that the survey respondent will leave their job (46). When the total score of the survey respondents' Turnover intention ≤ 6, it suggests a very low Turnover intention; 6 < total score ≤ 12 suggests a low Turnover intention; 12 < total score ≤ 18 suggests a high Turnover intention; >18 suggests a very high Turnover intention (47), and the Cronbach′s α coefficient for the present study was 0.95.

#### 2.2.3 Post-traumatic stress disorder

PTSD was assessed using the Impact of Event Scale, which is primarily used to measure psychological stress responses after exposure to stressful or traumatic events and is one of the earliest self-rating scales for PTSD. The scale includes 3 dimensions intrusion response, avoidance response, and hypervigilance, with a total of 22 entries. A 5-point Likert scale was adopted, and each entry was scored 0 (not at all) to 4 (always present), with higher scores indicating more severe symptoms (48); a total score of ≧33 was the critical value, indicating a high degree of suspicion of PTSD positivity; a total score of 22–32 indicated that a subclinical level of PTSD was reached, and the Cronbach′s α coefficient for the present study was 0.982 (49).

#### 2.2.4 Presenteeism

Presenteeism was measured using the Stanford Presenteeism Scale, which is divided into two dimensions: the work process impact dimension and the work outcome impact dimension, i.e., whether or not the work was completed for health reasons. The scale consists of six questions scored on a Likert 5 scale, with “1” being “Strongly Disagree”, “2” being “Disagree” “1” is “Strongly Disagree”, “2” is “Disagree”, “3” is “Unsure”, “4” is “Agree”. “5” is “Strongly Agree”, and entries 5 and 6 are reverse scored, with a total score of 6–30 (50). Higher scores indicate more serious productivity losses due to health reasons, and the Cronbach's α coefficient in this study was 0.922 (51).

#### 2.2.5 Social support

The social support used was Social Support Rating Scale (SSRS) was compiled by Shuiyuan Xiao based on the domestic situation and is suitable to be used as an instrument for this study, which is divided into three dimensions subjective support, objective support, and utilization of social support, with a total of 10 entries and scores ranging from 13 to 70 points (52). The sum of the scores for entries 1, 3, 4, and 5 is the subjective support score; the sum of the scores for entries 2, 6, and 7 is the objective support score; and the sum of the scores for entries 8, 9, and 10 is the support utilization score (53). The sum of the scores of the ten entries is the total score of social support, and the higher the score, the higher the level of social support, and the Cronbach's α coefficient of this study is 0.830.

### 2.3 Ethical clearance and data collection

Nursing staff were informed of the purpose and significance of this study, the voluntary nature of participation, and confidentiality. In addition, we also clearly communicated the purpose and potential outcomes of the PTSD and turnover intention assessment to the participants during the informed consent process. Participants have the right to withdraw at any time without any consequences. If participants showed significant distress or potential signs of PTSD, we provide information regarding access to a mental health professional or services. This study was conducted in April 2023 through the Questionnaire Star Online Survey Platform (https://www.wjx.cn), an online and open access survey platform spread through the social network mobile application WeChat. Firstly, the researcher contacted the relevant person in charge of the nursing department of the hospital to explain the purpose of the study and the precautions to be taken and obtained the consent, then the QR code was distributed through WeChat to the study subjects who met the inclusion and exclusion criteria to fill in the information, in order to ensure the accuracy of the information filled in, before filling in the information, it was filled in by the researcher with a unified instruction, and each ID was set to be filled out only once, and each question was mandatory. In order to ensure the completeness of the data, the questionnaire will be submitted only after all the questions have been filled. After the questionnaire is completed, it will be checked by the researcher to ensure the quality of the questionnaire. This study was approved by the Research Ethics Committee of Xuzhou Medical University (number: XZHMU-2023042).

### 2.4 Data analysis

Data analysis in this study was carried out using SPSS software and AMOS 23.0. Firstly, the mean and standard deviation were used for continuous variables, and conforming to a normal distribution (54), categorical variables were expressed as frequencies or percentages. Second, independent samples *t*-tests or one-way ANOVA were used to compare the difference between Turnover intention and general demographic information. Then, Pearson correlation analyses were used to analyze the relationship between important continuous variables (Turnover intention, PTSD, presenteeism, social support). In addition, a hierarchical multiple regression analysis was used to explore the factors influencing the Turnover intention of the job by including variables with *P* < 0.05 in the general information for a univariate analysis. Finally, we constructed a model with Turnover intention on the job as the dependent variable, PTSD as the independent variable, and social support and presenteeism as the mediator variables, and used AMOS 23.0 to further test the relationship between Turnover intention on the job, PTSD, social support, and presenteeism, and bootstrap (5,000 replications) method to test the mediating effect at the level of α = 0.05 (55). Model fit was assessed using the following metrics: χ2/df value < 5, goodness-of-fit index (GFI), Tucker-Lewis index (TLI), incremental fit index (IFI), and comparative fit index (CFI) all ≥0.90, and root mean square error of approximation (RMSEA) < 0.08 (56).

## 3 Results

### 3.1 Participant characteristics

A total of 2,513 nursing staff were included in this study, of which 3.9% were male and 96.1% were female, 81.4% were aged 25–44, 83.6% had a bachelor's degree, 16.2% of the nurses reported a history of alcohol consumption, 14.4% had taken a sedative and sleeping drugs, 63.5% had worked 8–12 h, 90.4% had cared for patients with COVID-19 patients, 97.9% of the nurses had contracted the novel coronavirus, the differences in the propensity to resign are statistically significant with respect to nursing staff age, department, specific hospital departments, marital status, history of alcohol consumption, use of sedative and hypnotic medications, employment status, years of experience, average working hours, whether they have provided care to COVID-19-infected patients, the outcomes of COVID-19-infected patients they cared for, the number of times they cared for COVID-19-infected patients, monthly income (Table 1).

**Table 1:** Univariate analysis of the demographic characteristics of the participants and factors related to turnover intention (*N* = 2,513).

**Variable**	**N (%)**	**Turnover intention**		
		**Mean (SD)**	***t*** **/** ***F***	***P***
**Gender**			1.41	0.16
Male	99 (3.9%)	2.19 (0.92)		
Female	2,414 (96.1%)	2.06 (0.88)		
**Age (years old)**			44.01	< 0.01
< 25	269 (10.7%)	2.05 (0.88)		
25–44	2,046 (81.4%)	2.12 (0.88)		
>44	198 (7.9%)	1.52 (0.72)		
**Department**			8.58	< 0.01
Outpatient department	251 (10.0%)	1.85 (0.84)		
Emergency department	187 (7.4%)	2.08 (0.88)		
Inpatient department	2,075 (82.6%)	2.09 (0.88)		
**Specific hospital departments**			4.45	< 0.01
Internal medicine	843 (33.6%)	2.08 (0.87)		
Surgery	570 (22.7%)	2.08 (0.90)		
Pediatrics	144 (5.7%)	2.13 (0.95)		
Obstetrics and gynecology	274 (10.9%)	2.05 (0.85)		
ICU	247 (9.8%)	2.21 (0.88)		
The emergency department	148 (5.9%)	2.05 (0.84)		
Other	287 (11.4%)	1.84 (0.85)		
**Education background**			0.91	0.40
Below undergraduate level	394 (15.7%)	2.02 (0.88)		
Undergraduate	2,101 (83.6%)	2.08 (0.88)		
Master degree or above	18 (0.7%)	2.18 (0.71)		
**Professional title**			32.90	< 0.01
Junior (nurse, nurse practitioner)	1,219 (48.5%)	2.11 (0.88)		
Intermediate (nurse-in-charge)	977 (38.9%)	2.14 (0.88)		
Senior (director of nurses, deputy director of the nurse practitioner)	317 (12.6%)	1.70 (0.79)		
**The only child**			−0.69	0.49
Yes	483 (19.2%)	2.04 (0.88)		
No	2,030 (80.8%)	2.07 (0.88)		
**Marital status**			3.53	0.03
Married	1,667 (66.3%)	2.03 (0.89)		
Unmarried	803 (32.0%)	2.13 (0.86)		
Divorced or widowed	43 (1.7%)	2.05 (0.91)		
**History of alcohol consumption**			3.94	< 0.01
Yes	407 (16.2%)	2.22 (0.90)		
No	2,106 (83.8%)	2.04 (0.87)		
**Take sedatives and sleepers**			7.75	< 0.01
Yes	362 (14.4%)	2.39 (0.86)		
No	2,151 (85.6%)	2.01 (0.87)		
**Form of employment**			44.92	< 0.01
Formal staffing	592 (23.6%)	1.79 (0.82)		
System of contract	1,787 (71.1%)	2.17 (0.88)		
Personnel agency	134 (5.3%)	1.94 (0.87)		
**Years of working (years)**			23.99	< 0.01
≤ 1	108 (4.2%)	1.89 (0.82)		
1–4	489 (19.5%)	2.13 (0.87)		
5–10	915 (36.5%)	2.20 (0.87)		
11–20	732 (29.1%)	2.04 (0.88)		
>20	269 (10.7%)	1.63 (0.81)		
**Average working hours per day (hours)**			60.74	< 0.01
≤ 8	682 (27.1%)	1.78 (0.78)		
8–12	1,594 (63.5%)	2.15 (0.89)		
≥12	237 (9.4%)	2.31 (0.91)		
**Participated in the province or city outbreak aid projects**			0.25	0.80
Yes	1,599 (63.6%)	2.07 (0.87)		
No	914 (36.4%)	2.06 (0.89)		
**Cared for COVID-19 patients**			4.00	< 0.01
Yes	2,272 (90.4%)	2.09 (0.88)		
No	241 (9.6%)	1.85 (0.84)		
**Outcomes for this COVID-19 patient**			13.16	< 0.01
Cured and discharged from hospital	1,263 (55.6%)	2.01 (0.89)		
Improved and discharged	809 (35.6%)	2.14 (0.85)		
Transfer to intensive care unit	100 (4.4%)	2.33 (0.88)		
Death	100 (4.4%)	2.46 (0.94)		
**Infected with the novel coronavirus**			2.77	0.01
Yes	2,460 (97.9%)	2.07 (0.88)		
No	53 (2,1%)	1.74 (0.79)		
**Infected with several new coronaviruses**			10.42	< 0.01
< 2	1,216 (49.4%)	1.99 (0.87)		
≥2	1,244 (50.6%)	2.15 (0.87)		
**Monthly income (yuan)**			6.02	< 0.01
1,000–3,000	154 (6.1%)	2.29 (0.99)		
3,001–5,000	626 (24.9%)	2.14 (0.87)		
5,000–10,000	1,410 (56.1%)	2.02 (0.87)		
More than 10,000	323 (12.9%)	2.04 (0.89)		

**Figure 2:**
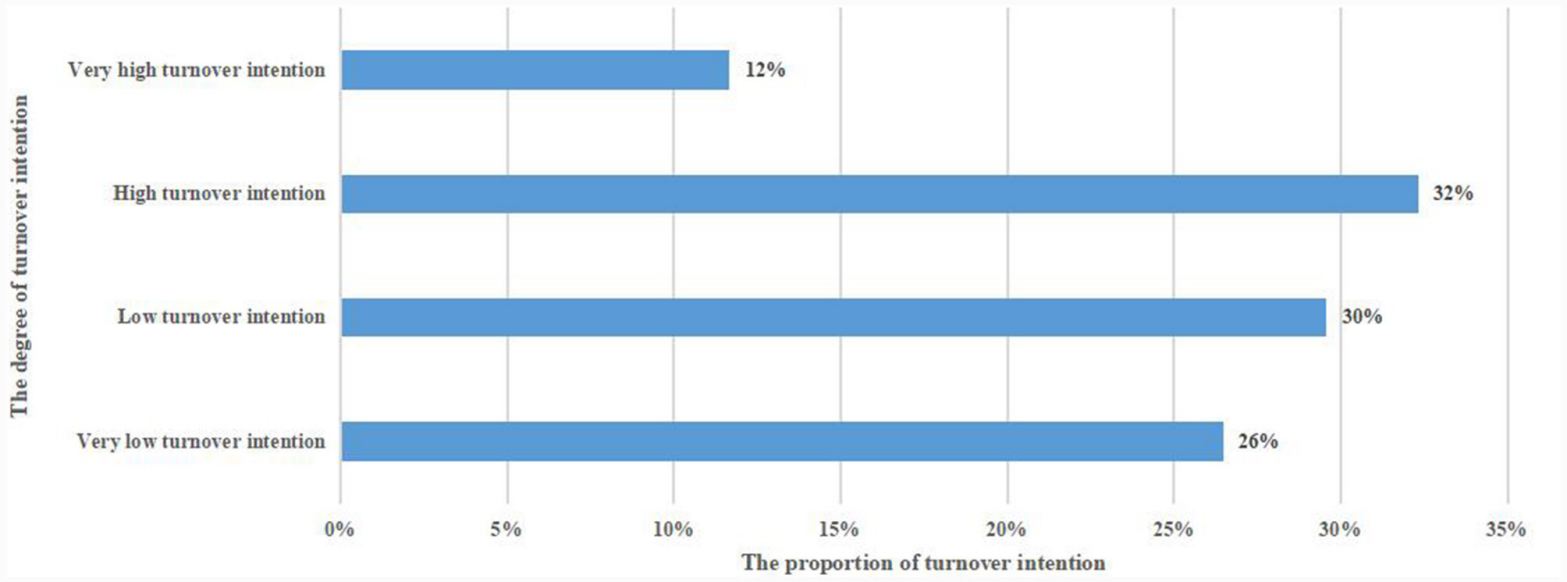
Turnover intention in nurses in the post-pandemic era.

In addition, there was a high Turnover intention in 32% of the nurses in this survey, and a very high Turnover intention in 12% of the nurses in the survey. Detailed results are shown in Figure 2, and the positivity rate for PTSD was 41%.

### 3.2 Correlation analysis

Pearson correlation analyses for Turnover intention, PTSD, social support, and presenteeism are shown in Table 2, where Turnover intention was negatively correlated with the dimensions of social support (*p* < 0.01), whereas Turnover intention was positively correlated with PTSD and presenteeism (*p* < 0.01).

**Table 2:** Correlation analysis between the various variables (*N* = 2,513).

**Relevance**	**Mean**	**SD**	**1**	**2**	**3**	**4**	**5**	**6**	**7**	**8**	**9**	**10**	**11**	**12**	**13**	**14**	**15**
1. Turnover intention	2.07	0.88	1														
2. The possibility of quitting your current job	2.07	0.94	0.958^**^	1													
3. Looking for other work motivation	2.05	0.89	0.945^**^	0.873^**^	1												
4. The possibility of external work	2.08	0.95	0.950^**^	0.872^**^	0.857^**^	1											
5. Social Support	1.28	0.28	−0.276^**^	−0.290^**^	−0.255^**^	−0.243^**^	1										
6. Subjective support	2.93	0.66	−0.237^**^	−0.251^**^	−0.214^**^	−0.211^**^	0.904^**^	1									
7. Objective support	3.08	0.71	−0.198^**^	−0.206^**^	−0.192^**^	−0.171^**^	0.689^**^	0.495^**^	1								
8. Support availability	0.40	0.15	−0.247^**^	−0.258^**^	−0.227^**^	−0.217^**^	0.785^**^	0.526^**^	0.447^**^	1							
9. Post-traumatic stress disorder	1.32	0.93	0.468^**^	0.444^**^	0.463^**^	0.434^**^	−0.192^**^	−0.141^**^	−0.164^**^	−0.194^**^	1						
10. Factor of invasion	0.70	0.71	0.399^**^	0.377^**^	0.394^**^	0.362^**^	−0.135^**^	−0.103^**^	−0.122^**^	−0.130^**^	0.869^**^	1					
11. Factor of avoidance	0.55	0.70	0.414^**^	0.396^**^	0.404^**^	0.381^**^	−0.175^**^	−0.132^**^	−0.143^**^	−0.178^**^	0.890^**^	0.862^**^	1				
12. High wake-up factor	0.56	0.70	0.441^**^	0.420^**^	0.428^**^	0.409^**^	−0.197^**^	−0.155^**^	−0.153^**^	−0.195^**^	0.890^**^	0.864^**^	0.902^**^	1			
13. Presenteeism	2.59	0.99	0.381^**^	0.372^**^	0.367^**^	0.345^**^	−0.101^**^	−0.075^**^	−0.095^**^	−0.093^**^	0.613^**^	0.610^**^	0.580^**^	0.609^**^	1		
14. Work process impact	2.47	1.09	0.430^**^	0.426^**^	0.413^**^	0.392^**^	−0.162^**^	−0.127^**^	−0.131^**^	−0.154^**^	0.643^**^	0.591^**^	0.582^**^	0.617^**^	0.940^**^	1	
15. Work result impact	2.84	1.16	0.180^**^	0.164^**^	0.178^**^	0.159^**^	0.043^*^	0.046^*^	−0.001	0.048^*^	0.364^**^	0.428^**^	0.369^**^	0.376^**^	0.748^**^	0.511^**^	1

^**^At the 0.01 level (two-tailed), the correlation was significant, and ^*^ at the 0.05 level (two-tailed), the correlation was significant.

### 3.3 Associations between variables

**Figure 3:**
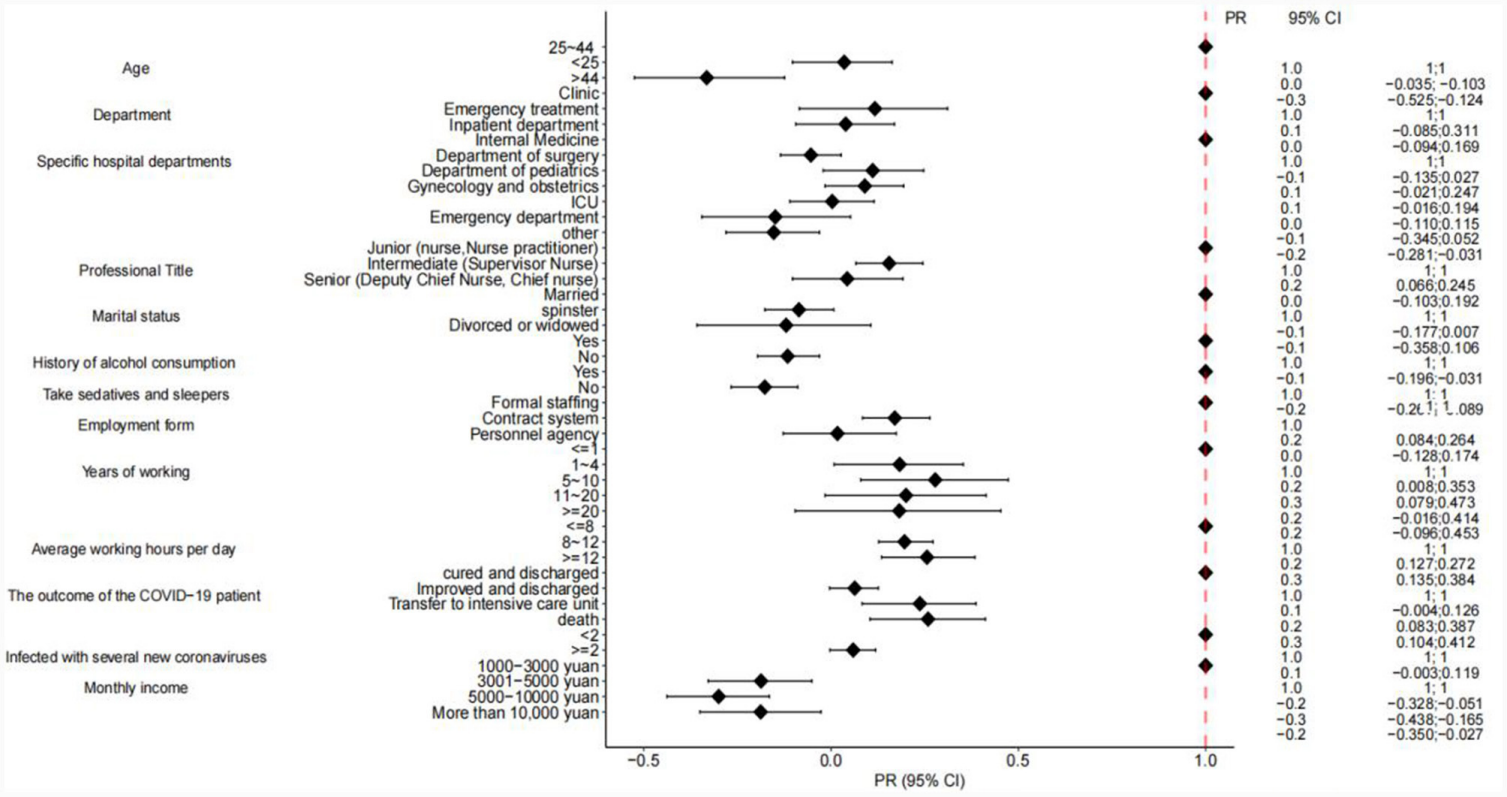
Impact of each subgroup on turnover intention.

Table 3 presents the results of the multiple regression analyses; first, statistically significant variables were included in the general information (e.g., age, department, professional title, years of experience, etc.) into the model, explaining 15.3% of the variance in the Turnover intention. Next, we included PTSD in the model again, explaining 33.1% of the variance. Finally, we included social support and presenteeism into the model again, explaining 35.7% of the variance. Figure 3 shows that the effects of nurses' Turnover intention is significant on different age groups, alcohol consumption, sedative sleeping drugs, contract system, working hours < 10 years, working hours more than 8 h, and monthly income.

**Table 3:** Hierarchical linear regression analysis of turnover intention of nurses in post-pandemic era (*N* = 2,513).

**Variables**	**Turnover intention**		
	**Step 1 (**β**)**	**Step 2 (**β**)**	**Step 3 (**β**)**
**Age (reference:25–44)**			
< 25	−0.008	0.007	0.035
>44	−0.325^**^	−0.33^**^	−0.332^**^
**Department (reference: clinic)**			
Emergency treatment	0.091	0.110	0.117
Inpatient department	0.002	0.049	0.039
**Specific hospital departments (reference: internal medicine)**			
Department of surgery	−0.037	−0.042	−0.054
Department of pediatrics	0.155^*^	0.117	0.111
Gynecology and obstetrics	0.077	0.082	0.090
ICU	−0.023	−0.002	0.003
Emergency department	−0.164	−0.141	−0.149
other	−0.22^**^	−0.174^**^	−0.153^*^
**Professional title [reference: junior (nurse, nurse practitioner)]**			
Intermediate (supervisor nurse)	0.159^**^	0.137^**^	0.155^**^
Senior (deputy chief nurse, chief nurse)	−0.024	0.003	0.043
**Marital status (reference: married)**			
Spinster	0.001	0.026	−0.086
Divorced or widowed	0.011	−0.056	−0.120
**History of alcohol consumption (ref: yes)**			
No	−0.127^**^	−0.119^**^	−0.116^**^
**Take sedatives and sleepers (reference: yes)**			
No	−0.335^***^	−0.217^***^	−0.177^***^
**Employment form (reference: formal staffing)**			
Contract system	0.183^**^	0.160^**^	0.170^***^
Personnel agency	0.057	−0.018	0.017
**Years of working (reference:** ≤ **1)**			
1–4	0.254^*^	0.178^*^	0.183^*^
5–10	0.300^**^	0.285^**^	0.278^**^
11–20	0.236	0.206	0.200
≥20	0.253	0.171	0.182
**Average working hours per day (reference:** ≤ **8 h)**			
8–12	0.325^***^	0.207^***^	0.196^***^
≥12	0.498^***^	0.321^***^	0.256^***^
**The outcome of the COVID**−**19 patient (reference: cured**			
**and discharged)**			
Improved and discharged	0.154^***^	0.086^*^	0.063
Transfer to intensive care unit	0.296	0.269^**^	0.237^**^
Death	0.347^**^	0.285^***^	0.259^**^
**Infected with several new coronaviruses (reference:**<**2)**			
≥2	0.139^***^	0.074^*^	0.059
**Monthly income (reference: 1,000–3,000 yuan)**			
3,001–5,000 yuan	−0.170^*^	−0.196^**^	−0.187^**^
5,000–10,000 yuan	−0.357^***^	−0.312^***^	−0.300^***^
More than 10,000 yuan	−0.348^***^	−0.203^*^	−0.188^*^
Post-traumatic stress disorder		0.415^***^	0.313^***^
Social Support			−0.502^***^
Presenteeism			0.119^***^
*F*	11.288^***^	30.065^***^	32.028^***^
*R* ^2^	0.153	0.331	0.357
Δ*R*^2^	0.153	0.331	0.357

^*^P < 0.05, ^**^P < 0.01, ^***^P < 0.001.

### 3.4 Modeling of mediating effects

**Figure 4:**
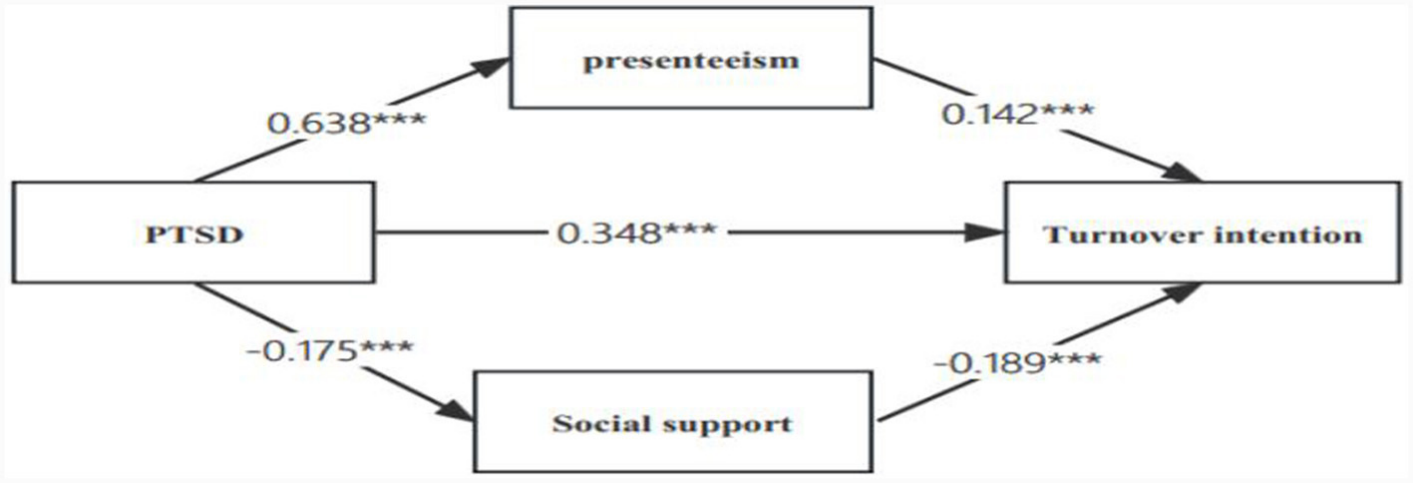
Mediating effect of social support and presentation on the relationship between PTSD and turnover intention PTSD, post-traumatic stress disorder; ***P << 0.011.

Table 4 shows the results of analyzing the models of social support and presenteeism on the Turnover intention of the job and PTSD, which were estimated using the maximum likelihood method, and the models were fitted with good indicators (CMIN/DF = 2.411, RMSEA = 0.024, IFI = 0.999, TLI = 0.996 CFI = 0.999). Turnover intention (β = 0.348, *P* < 0.001), PTSD was a positive predictor of presenteeism (β = 0.638, *P* < 0.001); in the PTSD → presenteeism → Turnover intention pathway, there was an indirect effect of 0.086, *P* < 0.001, with an effect share of 19%. In the PTSD → social support → Turnover intention pathway, the indirect effect was 0.031, *P* < 0.001, and the effect share was 7%. Social support and presenteeism played a mediating role in PTSD and intention to leave the job, as shown in the model diagram in Figure 4. In addition, we also compared the difference between the two paths, with an effect of 0.086 for path 1 and 0.031 for path 2, a difference of 0.055.

**Table 4:** Total, direct, and indirect effects of PTSD on turnover intention (*N* = 2,513).

**Effect**	**Paths**	**Standardized estimates**	**95%CI**
Direct	PTSD → Presenteeism	0.638^***^	(0.603, 0.670)
	PTSD → Social support	−0.175^***^	(−0.213, −0.134)
	Presenteeism → Turnover intention	0.142^***^	(0.094, 0.191)
	Social support → Turnover intention	−0.189^***^	(−0.224, −0.156)
	PTSD → Turnover intention	0.348^***^	(0.296, 0.398)
Indirect	PTSD → Presenteeism → Turnover intention	0.086^***^	(0.056, 0.118)
	PTSD → Social support → Turnover intention	0.031^***^	(0.023, 0.042)
Total	PTSD → Turnover intention	0.472^***^	(0.415, 0.483)

PTSD, post-traumatic stress disorder; a 95% bootstrap confidence interval that does not include zero means an effect is significant.

^***^P < 0.001.

## 4 Discussion

To the best of our knowledge, this study is the first to examine the structural relationship between PTSD and the turnover intention among Chinese nurses in the post-epidemic era. This study found that in the post-epidemic era, the level of PTSD among nurses decreased compared to those of PTSD during the pandemic (57). One possible explanation for this result is that in the post-epidemic era, nurses may be faced with relatively less or more controlled traumatic factors or stressors, with a reduction compared to during the pandemic (58). This may be influenced by a range of factors, some of which may include: the post-epidemic era may mark the easing of the epidemic and the gradual return of society to normal. This situational change may have reduced the tension and uncertainty faced by the nurses. This study also found that nurses with high or above turnover intentions were less in the post-epidemic era than during the outbreak study by Melanie Lavoie-Tremblay et al. (59). The possible reason is that during outbreaks, nurses may be at risk of infection, leading to concerns about personal safety. After the outbreak, as cases decrease and the vaccine spreads, nurses may feel safer, leaving less incentive to find other job opportunities (60). Moreover, during the outbreak, nurses may play a critical role in providing critical medical services to patients. This experience may have improved nurses' identity with their profession and made them more willing to stay in their current position (61). But future research should also understand the deeper causes.

Our study found that presenteeism as a mediator between PTSD and willingness to leave, with a positive correlation between presenteeism and turnover tendency, and PTSD. Ning et al. (33) surveyed 703 primary health care workers in Jilin Province, China, and showed a significant positive relationship between presenteeism and turnover tendency. Liao et al. (35) investigated 836 medical service workers in Hunan Province, China, and showed a positive correlation between PTSD and presenteeism. The above findings are consistent with our findings, because in the post-epidemic era, with the relaxation of epidemic prevention and control policies, prolonged excessive workload, psychological stress, and coming to work with illness may lead to an increased risk of PTSD, and may also prompt nurses to consider leaving their jobs in search of a better health and work balance (14, 18). Our study also found a significant correlation between average daily working hours and turnover intention among nurses. Therefore, nursing managers and healthcare organizations should improve the allocation of human resources by rationalizing shift scheduling and improving the allocation of human resources. There is one study showing that some evidence-based interventions can change the physical and mental health and psychological status of nurses and highlight the importance of taking time to exercise and balance work and life (62).

This study that social support plays a protective role in PTSD and Turnover intention is of great importance to nurses in the context of post-epidemic era, which can be explained by resource conservation theory (63), social support can be considered a psychological resource. Traumatic events can have a serious impact on an individual's mental health, leading to anxiety, depression, and exacerbation of symptoms (64). Social support can provide emotional, informational, and substantive support to help individuals cope with negative emotions after trauma (65). However, when individuals have adequate social support, they may feel more emotional support and encouragement, which may enhance their ability to cope with work difficulties, which may make them more willing to stay in their current job rather than choose to leave (66). However, during the outbreak, Tatsuno et al. (67) conducted a cross-sectional survey of ICU nurses in Japan, and the findings showed no relationship between social support and PTSD. This varies from our findings, where social support was not associated with psychological distress in the early stages and later stages in Cook and Bickman (68). Therefore, in the late stage, social support can affect the psychological trauma. Because our study was a survey in the post-epidemic era, there is a relationship between nursing staff social support and PTSD. Therefore, in the post-epidemic era, our study has the following insights for medical institutions or managers. First, medical institutions should continue to implement workplace health promotion programs and occupational health surveillance and reduce the psychological trauma of nurses, for early diagnosis and prevention (69). Second, support for nurses should be increased, including family support and assistance, and their income should be increased (63). Finally, develop rationalized working hours to reduce the workload of nurses and avoid overwork.

We also acknowledge that this study also has some limitations. Firstly, the population of this study was mainly female and originated from tertiary hospitals, which may limit the findings in terms of generalization, secondly, this study was a cross-sectional study, so the findings do not explain the causal relationship between the variables. Finally, this study did not consider such factors as nurse work stress, therefore, future studies should further incorporate these factors to get a more comprehensive understanding of nurse turnover intention. It is noteworthy that PTSD is dynamic and a future longitudinal study design should also be used to explore the impact of the post-pandemic period on nurses. Alternatively, future research could explore the moderating role of mediating variables to explain the effect of PTSD on the propensity to leave a job.

Despite the limitations of this study, our research further explored the relationship between PTSD and Turnover intention in the post-pandemic era. Our findings also provide an understanding of the psychological dilemmas faced by nurses during post-pandemic era, which can help provide them with more effective mental health support and interventions.

## 5 Conclusions

This study found that post-traumatic stress disorder and turnover intention remained high among nurses in the post-epidemic era. Social support and presenism play a mediating role between PTSD and willingness to leave. Nurses' turnover intention was significantly correlated with factors such as professional title and average working hours per day. In the post-pandemic period, managers and health care institutions should increase their mental health support and attention to nurses and provide the counseling and support services necessary to reduce the psychological stress that nurses may face. Furthermore, healthcare institutions should encourage the establishment of active social support networks, enhance collaboration and support among colleagues, and provide nurses with moderate work breaks and self-care opportunities to prevent overdeveloped attendance, which can lead to adverse consequences.

## Data availability statement

The original contributions presented in the study are included in the article/supplementary material, further inquiries can be directed to the corresponding author.

## Ethics statement

Written informed consent was obtained from the individual (s) for the publication of any potentially identifiable images or data included in this article.

## Author contributions

JZ: Data curation, Methodology, Writing—original draft, Writing—review & editing. XY: Data curation, Methodology, Software, Supervision, Writing—original draft. XZ: Conceptualization, Funding acquisition, Investigation, Resources, Software, Writing—original draft. YL: Conceptualization, Investigation, Methodology, Writing—original draft, Writing—review & editing. MengsL: Methodology, Software, Writing—original draft, Writing—review & editing. YF: Formal analysis, Methodology, Project administration, Validation, Writing—original draft, Writing—review & editing. MengjL: Conceptualization, Investigation, Writing—original draft, Writing—review & editing. MW: Data curation, Formal analysis, Investigation, Methodology, Writing—original draft, Writing—review & editing.
